# Glass Transition, Liquid Dynamics, and Thermal Degradation in 2D Hybrid Halide Perovskites

**DOI:** 10.1002/smll.202500311

**Published:** 2025-04-02

**Authors:** Owain S. Houghton, Chumei Ye, Alison C. Twitchett‐Harrison, Siân E. Dutton, Thomas D. Bennett, A. Lindsay Greer

**Affiliations:** ^1^ Department of Materials Science & Metallurgy University of Cambridge 27 Charles Babbage Road Cambridge CB3 0FS UK; ^2^ Cavendish Laboratory University of Cambridge J. J. Thomson Avenue Cambridge CB3 0HE UK; ^3^ WPI Advanced Institute for Materials Research Tohoku University Sendai 980–8577 Japan

**Keywords:** calorimetry, degradation, glass formation, hybrid perovskites, liquid dynamics

## Abstract

2D hybrid organic–inorganic perovskites (2D HOIPs) are of interest for optoelectronic and phase‐change applications. Using ultra‐fast (flash) differential scanning calorimetry (FDSC), this study shows the 2D HOIPs (*S*‐Cl‐MBA)_2_PbI_4_ and (*R*‐Cl‐MBA)_2_PbBr_4_ (Cl‐MBA referring to 4‐chloro‐α‐methylbenzylamine) form a glass on cooling. Both show evidence of a liquid‐to‐glass transition during quenching from the liquid state; on reheating, a glass‐to‐liquid transition is followed by crystallization and melting. Using continuous heating in FDSC, the temperature dependence of the liquid viscosity of (*S*‐Cl‐MBA)_2_PbI_4_ is characterized. The kinetic fragility of the liquid is similar to that of bulk metallic glass‐formers and significantly lower than that of organic and phase‐change chalcogenide liquids. On cooling the liquid, glass formation is first impeded by thermal degradation, then crystallization. The stages of thermal degradation can be related to known mechanisms. This study highlights the reduced glass‐transition temperature and the liquid fragility as key parameters in guiding the optimization of 2D HOIP compositions for targeted applications.

## Graphical abstract

Glass formation in 2D hybrid halide perovskites can be achieved by rapid quenching of melted material using ultra‐fast (flash) differential scanning calorimetry (FDSC). As‐quenched (*S*‐Cl‐MBA)_2_PbI_4_ and (*R*‐Cl‐MBA)_2_PbBr_4_ both show evidence of a reversible glass transition on heating. Continuous heating and cooling studies are used to study the dynamics of liquid and degradation of (*S*‐Cl‐MBA)_2_PbI_4_.

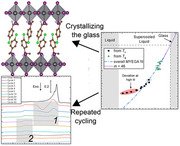

## 1 Introduction

Hybrid organic–inorganic perovskites (HOIPs) are promising semiconducting materials for ferroelectrics, light‐emitting diodes, laser devices, and photovoltaics.^[^ ^1^, ^2^, ^3^, ^4^, ^5^, ^6^, ^7^, ^8^, ^9^, ^10^, ^11^ ^]^ Perovskites have the general formula ABX_3_, exemplified by CaTiO_3_. HOIPs differ from conventional inorganic perovskites in that the A‐site cations are organic; in most cases, the B‐site ions remain metallic, and the X‐site cations are typically halides, though they can also be small molecules.^[^ ^12^ ^]^ The nature of the A‐site cation determines the dimensionality of the overall structure. For smaller A‐site cations, HOIPs have the same structure as conventional perovskites — a 3D crystal of corner‐sharing BX_6_‐type octahedral cages. With larger cations,^[^ ^13^ ^]^ the chemical stability increases, and the structure evolves into layers of linked BX_6_ octahedra separated by A‐site cations. These 2D HOIPs can be subdivided into Dion‐Jacobsen or Ruddlesden‐Popper (RP) phases according to the functionality of the A‐site cation.^[^ ^12^ ^]^

Melting and vitrifying HOIPs is of interest due to the differing properties of the crystalline and glassy states.^[^ ^14^, ^15^ ^]^ The ability to reversibly change phase, and the distinct optical responses of the glassy and crystalline states, makes them prospective materials for phase‐change memory, optical communication, and neuromorphic computing.^[^ ^13^ ^]^

The main challenge in forming such glasses via melt‐quenching is the need to melt the crystalline material and quench the liquid into the glassy state without significant thermal decomposition. Through careful choice of constituent molecules, several HOIPs have been synthesized that can be melted before they decompose.^[^ ^12^ ^]^

Several such HOIPs have since been shown to form a glass when quenched from the liquid.^[^ ^14^, ^15^, ^16^, ^17^, ^18^, ^19^ ^]^ Amorphous HOIPs have also been formed by ball‐milling or applying pressure to crystalline material.^[^ ^20^, ^21^, ^22^ ^]^ However, the nature of glass formation and the ability to cycle between glassy and crystalline states have received scant attention.

Ultra‐fast (flash) calorimetry, FDSC, has been used to study the glass‐forming ability and devitrification of chalcogenide,^[^ ^23^ ^]^ metallic,^[^ ^24^ ^]^ and polymeric systems,^[^ ^25^ ^]^ and more recently HOIPs.^[^ ^17^, ^19^ ^]^ With a small sample size, cooling rates as high as 4 × 10^4^ K s^−1^ and heating rates as high as 5 × 10^4^ K s^−1^, a wide range of thermal treatments can be achieved. If HOIPs are to be developed for applications dependent on the glassy phase, knowledge of glass‐forming ability and crystallization kinetics is necessary, especially at the high heating and cooling rates relevant for phase‐change applications.^[^ ^17^, ^19^, ^23^ ^]^

In the present work, FDSC and transmission electron microscopy (TEM) are used to study two Ruddlesden‐Popper chiral 2D HOIPs: (*S*‐Cl‐MBA)_2_PbI_4_ and (*R*‐Cl‐MBA)_2_PbBr_4_ (Cl‐MBA referring to 4‐chloro‐α‐methylbenzylamine). These HOIPs can melt before they decompose,^[^ ^12^ ^]^ but their glass‐forming ability has not yet been reported. We find that for each HOIP, a glass can be formed by melting and quenching. For (*S*‐Cl‐MBA)_2_PbI_4_, the non‐isothermal crystallization of the glass is characterized in order to probe the sequence of thermal degradation and the dynamics of the glassy and liquid states.

## 2 Results

### 2.1 Crystalline Structure of As‐Synthesized (*S*‐Cl‐MBA)_2_PbI_4_ and (*R*‐Cl‐MBA)_2_PbBr_4_

**Figure 1:**
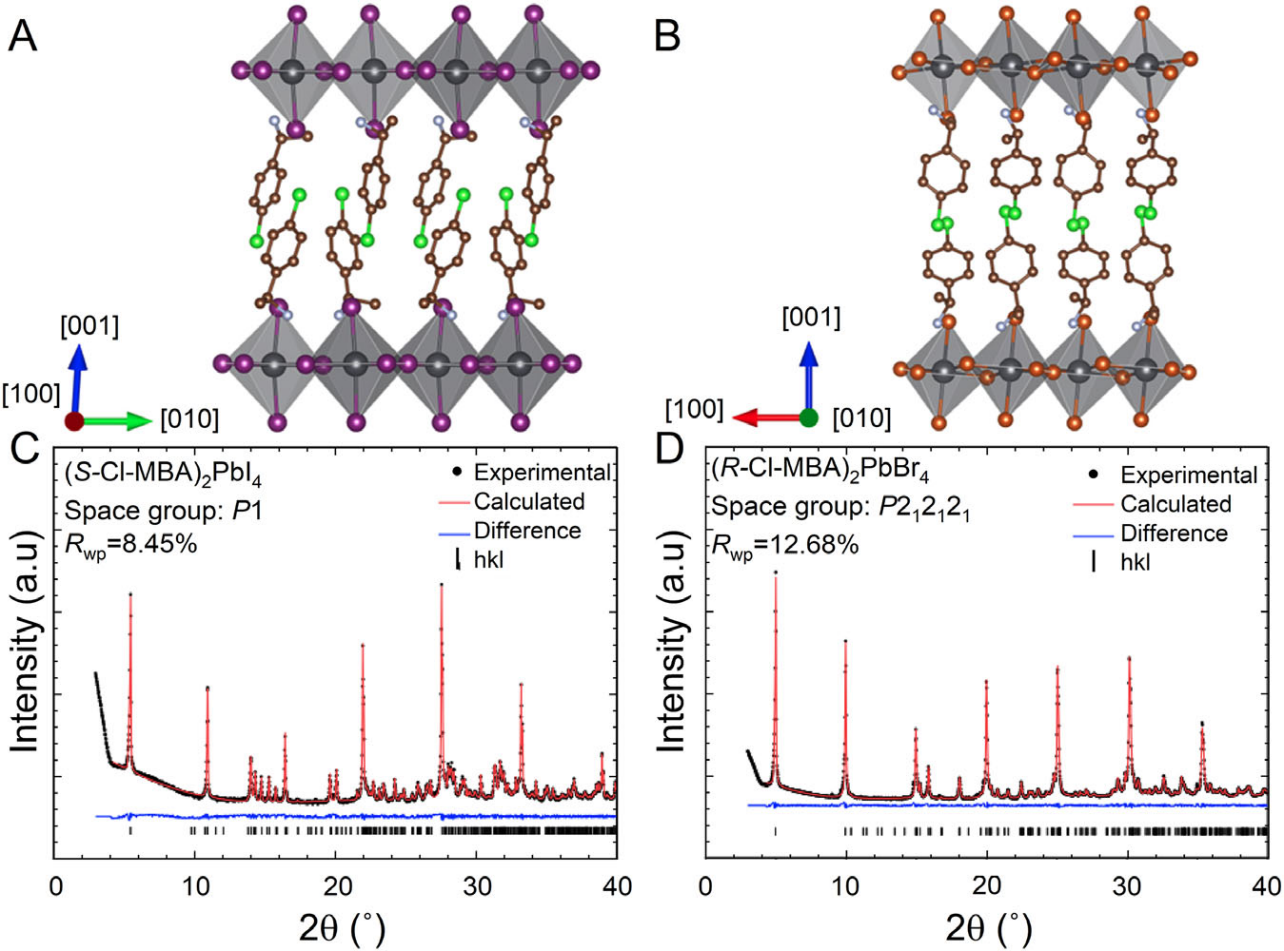
Structure of as‐synthesized, crystalline (*S*‐Cl‐MBA)_2_PbI_4_ and (*R*‐Cl‐MBA)_2_PbBr_4_. Schematic crystal structures of A) (*S*‐Cl‐MBA)_2_PbI_4_ and B) (*R*‐Cl‐MBA)_2_PbBr_4_. Each atom is colored according to the element it represents: Pb (black), I (purple), Br (orange), C (brown), N (light purple), and Cl (green). All H atoms are omitted for clarity. Pawley refinements (red lines) of the powder diffraction patterns for C) (*S*‐Cl‐MBA)_2_PbI_4_ and D) (*R*‐Cl‐MBA)_2_PbBr_4_ show a close fit to the experimental diffractogram (black dots). Permitted Bragg reflections for the identified phases are shown for Cu Kα radiation (1.5418 Å).

Crystalline (*S*‐Cl‐MBA)_2_PbI_4_ and (*R*‐Cl‐MBA)_2_PbBr_4_ were synthesized by slowly cooling an aqueous solution of stoichiometric PbX_2_ (X = I or Br) and the corresponding chiral amine, according to the method reported in refs. [26, 27]. Both crystals have a typical 2D RP phase structure, in which each single inorganic layer of corner‐sharing [PbX_6_]^4–^ octahedra is separated by a bilayer of chiral organic cations (**Figure** **1**A,B).

Pawley refinement of powder X‐ray diffraction patterns for each HOIP (Figure 1C,D) confirms that (*S*‐Cl‐MBA)_2_PbI_4_ crystallizes into a triclinic structure (space group: *P*1), while (*R*‐Cl‐MBA)_2_PbBr_4_ crystallizes into an orthorhombic structure (space group *P*2_1_2_1_2_1_). The refinements suggest both materials are phase‐pure. Crystallographic and refinement parameters are summarized in Tables S1 and S2 (Supporting Information).

### 2.2 Degradation Behavior of (*S*‐Cl‐MBA)_2_PbI_4_ and (*R*‐Cl‐MBA)_2_PbBr_4_

Thermogravimetric analysis (TGA) of each HOIP under an argon atmosphere suggests a two‐stage decomposition process (Figure S1, Supporting Information).^[^ ^12^, ^28^ ^]^ The onset of degradation (at *T*_d_) on heating at 10 K min^−1^ is ≈495 K for (*S*‐Cl‐MBA)_2_PbI_4_ and 500 K for (*R*‐Cl‐MBA)_2_PbBr_4_. For *S*‐Cl‐MBA)_2_PbI_4_, the first degradation stage that occurs from 495 to 640 K leads to a weight loss of 55.5 wt.%. The measured weight loss agrees with the theoretical mass loss value (55.2 wt.%) for the release of organic cations and the formation of the intermediate PbI_2_. Similar decomposition behavior was also observed in (*R*‐Cl‐MBA)_2_PbBr_4_, degrading to form intermediate PbBr_2_ at ≈600 K (Figure S1, Supporting Information). For both HOIPs, the second degradation stage occurs above 750 K with breakdown of the octahedral layers.^[^ ^12^, ^28^ ^]^

### 2.3 Glass‐Forming Ability of (*S*‐Cl‐MBA)_2_PbI_4_ and (*R*‐Cl‐MBA)_2_PbBr_4_

**Figure 2:**
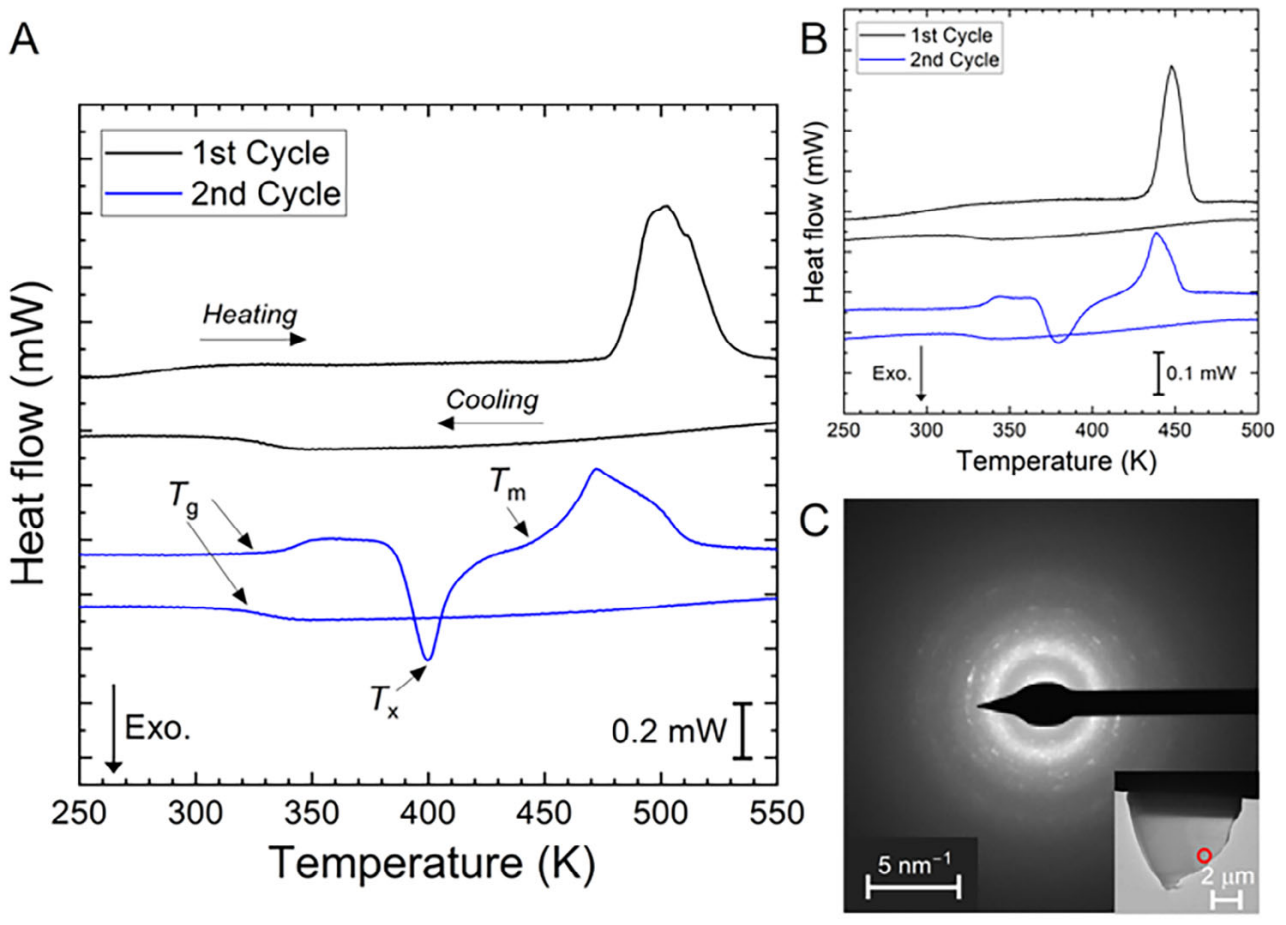
Glass formation in (*S*‐Cl‐MBA)_2_PbI_4_ and (*R*‐Cl‐MBA)_2_PbBr_4_. A) Thermograms of as‐synthesized (*S*‐Cl‐MBA)_2_PbI_4_ heated to 550 K and cooled at 1000 K s^−1^, and of a second heating/cooling at the same rate. B) The same as (A) but for (*R*‐Cl‐MBA)_2_PbBr_4_ heated to 500 K (rather than 550 K, to reduce the degree of thermal degradation). C) Selected‐area electron diffraction pattern of a lamella of (*S*‐Cl‐MBA)_2_PbI_4_ extracted by FIB‐SEM from a sample quenched at 5000 K s^−1^ (shown inset, in which the red circle highlights the region from which the diffraction pattern was taken).

FDSC thermograms of (*S*‐Cl‐MBA)_2_PbI_4_ and (*R*‐Cl‐MBA)_2_PbBr_4_ were obtained for the as‐synthesized material heated through melting and then quenched from the liquid at 1000 K s^−1^. Both show an endotherm during the initial heating run at 1000 K s^−1^, consistent with melting of the crystalline phase (**Figure** **2**A,B). On cooling, both samples show a step to a lower specific heat capacity just below 350 K. This step is subsequently reversed at a similar temperature on reheating at 1000 K s^−1^. The form of this calorimetric feature, and its reversibility, support its identification as the transition from liquid to glass on cooling and from glass to liquid on heating. The onset temperature *T*_g_ for the glass transition on heating at 1000 K s^−1^ is 338 K for (*S*‐Cl‐MBA)_2_PbI_4_ and 331 K for (*R*‐Cl‐MBA)_2_PbBr_4_.

On continued heating, for each HOIP, the glass transition is followed by an exotherm and an endotherm, presumed to correspond respectively to crystallization and to melting of the material. The temperature, *T*_x_, of the peak of the crystallization exotherm is 400 and 379 K, and the temperature, *T*_m_, of the onset of melting is 450 and 426 K for (*S*‐Cl‐MBA)_2_PbI_4_ and (*R*‐Cl‐MBA)_2_PbBr_4_, respectively.

For each HOIP, the melting endotherm following crystallization of the glass is at lower temperature than that in the initial thermograms of as‐synthesized samples. On first melting an FDSC sample, it flows to have a greater contact area with the chip. This improves the thermal contact between the sample and the chip during subsequent heating runs, and this may partly account for the shift in measured *T*_m_.

**Figure 3:**
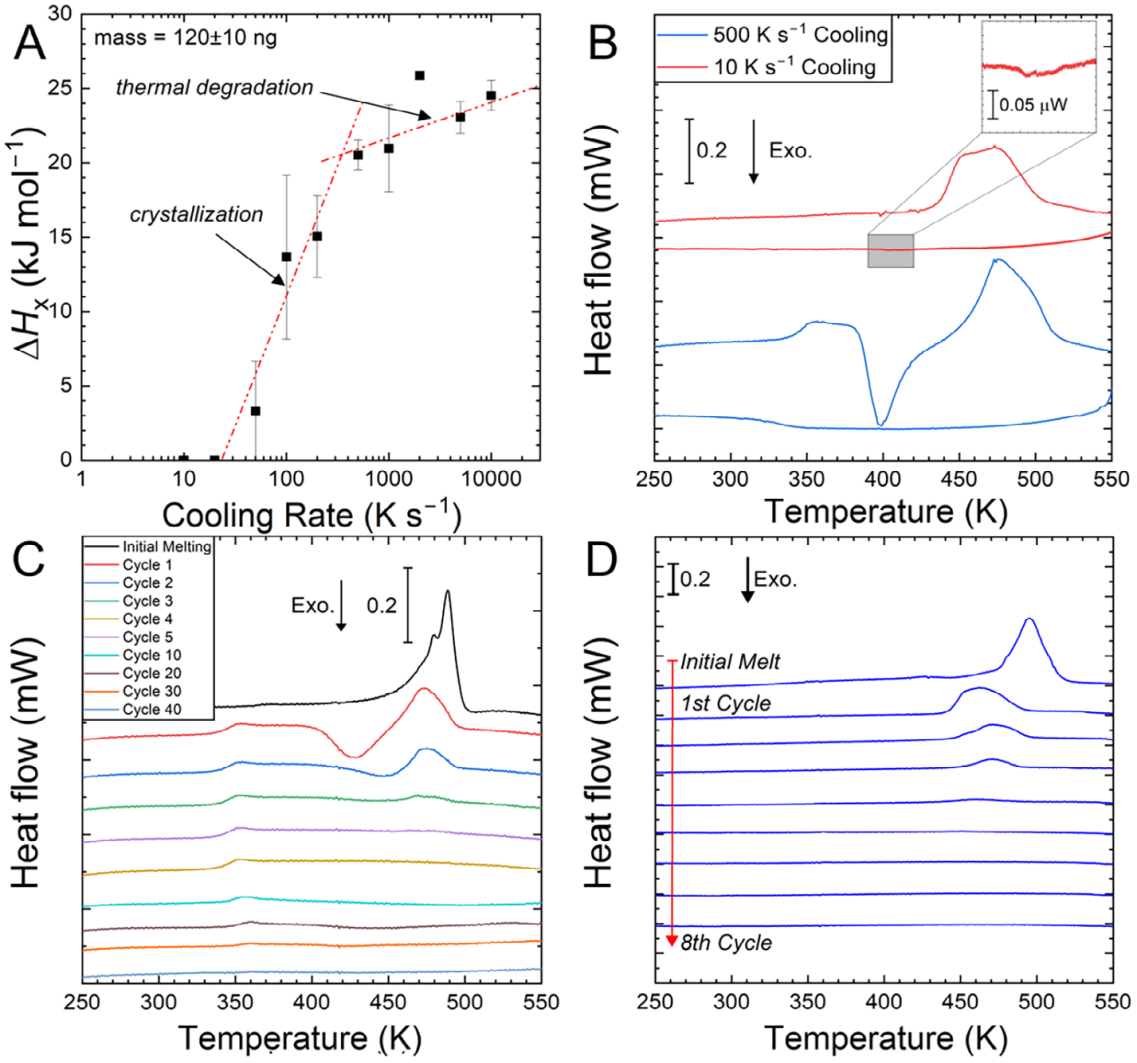
Thermal degradation of (*S*‐Cl‐MBA)_2_PbI_4_ during heating and cooling. A) Heat of crystallization measured on reheating at 1000 K s^−1^ as a function of the cooling rate at which the sample was formed from the liquid. For each data point, three samples were measured. B) Thermograms on reheating at 1000 K s^−1^ after cooling at 500 or 10 K s^−1^. The cooling curve at 10 K s^−1^ shows evidence of crystallization (inset), and the subsequent heating thermogram shows no glass transition nor crystallization. The cooling curve at 500 K s^−1^ shows a glass transition without crystallization. C) Thermograms of the heating segments during repeated heating and cooling at 1000 K s^−1^ for 40 cycles. The sample was stored for 9 months in a sealed container before testing. Results from a sample cut from the same batch immediately after synthesis are shown in Figure S2 (Supporting Information). D) Thermograms on heating at 1000 K s^−1^ for up to 9 cycles with intervening cooling at 10 K s^−1^.

It is possible, however, that the shift also indicates that the crystalline state formed on devitrification is metastable relative to (i.e., has a higher free energy than) the as‐synthesized crystalline state. The metastable state is taken to be a lower‐symmetry phase that subsequently transforms into a higher‐symmetry phase via inverse‐chain melting.^[^ ^29^, ^30^, ^31^ ^]^ The higher‐symmetry phase subsequently melts in the conventional sense. For each HOIP, the melting endotherm is asymmetric, suggesting two overlapping peaks. This is best seen when the cooling rate used to form the glass, and heating rate used to characterize that sample are relatively low (**Figure** **3**B). The start of conventional melting before inverse chain melting is complete may lead to the complex profile of the endotherm.

For both (*S*‐Cl‐MBA)_2_PbI_4_ and (*R*‐Cl‐MBA)_2_PbBr_4_, when a glassy sample is quenched at 1000 K s^−1^ and then heated at the same rate, the heat of crystallization ∆*H*_x_ is 85% of the heat of melting ∆*H*_m_ (Figure 2A,B). This does not imply, however, that the quenched samples are 15% crystallized. For a fully glassy sample, ∆*H*_x_ is expected to be less than ∆*H*_m_ (for a typical metallic glass, ∆*H*_x_/∆*H*_m_≈0.4);^[^ ^32^ ^]^ this is because, over the temperature range between crystallization and melting, the liquid can have a significantly higher heat capacity than the crystal. This excess heat capacity of the liquid relative to the crystal results in a smaller enthalpy difference at *T*_x_ compared to *T*_m_.

The width of the supercooled liquid region ∆*T*_x_ (= *T*_x_ ‒ *T*_g_) is ≈62 K for (*S*‐Cl‐MBA)_2_PbI_4_ and ≈48 K for (*R*‐Cl‐MBA)_2_PbBr_4_. Increased ∆*T*_x_ indicates greater opportunity for processing a glass without the onset of crystallization. For this reason, (*S*‐Cl‐MBA)_2_PbI_4_ was chosen for further investigation. TEM was performed on a lamella of (*S*‐Cl‐MBA)_2_PbI_4_ extracted by focused ion beam (FIB) milling from a sample heated to 550 K at 1000 K s^−1^ and subsequently quenched at 5000 K s^−1^ using FDSC. The diffraction pattern (Figure 2C) suggests that the sample is mostly amorphous, but with a slight degree of crystallinity. The origin of the crystallization cannot be deduced from this work; it may occur during quenching, lamella preparation, or storage at room temperature (since room temperature is a high fraction of *T*_g_).

The glass‐forming ability of (*S*‐Cl‐MBA)_2_PbI_4_ was investigated by varying the cooling rate at which the liquid is quenched, after heating the as‐synthesized samples into the fully liquid state at the same rate, in the range 10–10^4^ K s^−1^). The enthalpy of crystallization ∆*H*_x_ was measured on reheating the as‐obtained glass at 1000 K s^−1^ (Figure 3A). In a study of this kind, it is expected that as the cooling rate is increased, the fraction of the sample that is glassy is higher, and consequently ∆*H*_x_ is higher. For cooling rates exceeding a critical value, the samples would be fully glassy and so show a maximum and invariant ∆*H*_x_. In the present case, there is a clear change of regime, indicating a critical cooling rate. Below 300 K s^‒1^, ∆*H*_x_ increases sharply with cooling rate as expected. Above 300 K s^−1^, however, ∆*H*_x_ does not level off, but continues to rise, albeit more slowly. This variation is attributed to partial decomposition of the HOIP,^[^ ^28^ ^]^ with more decomposition occurring during slower cooling. Crystallization on slow cooling can be observed only at much lower cooling rates (Figure 2B).

The extent of decomposition was explored by subjecting a sample to repeated heating through melting (up to 550 K) and cooling at 1000 K s^−1^ (i.e., well above the critical cooling rate at which the sample is fully glassy). Over the first few cycles, the crystallization and melting peaks progressively disappear, and after more cycles the glass transition disappears (Figure 3C). After 40 cycles, no thermal transitions are observed, indicating that the sample has fully degraded. The same effect is seen on cycling with the lower cooling rate of 10 K s^−1^ (when the sample completely crystallizes on cooling), but the degradation occurs over fewer cycles (Figure 3D).

**Figure 4:**
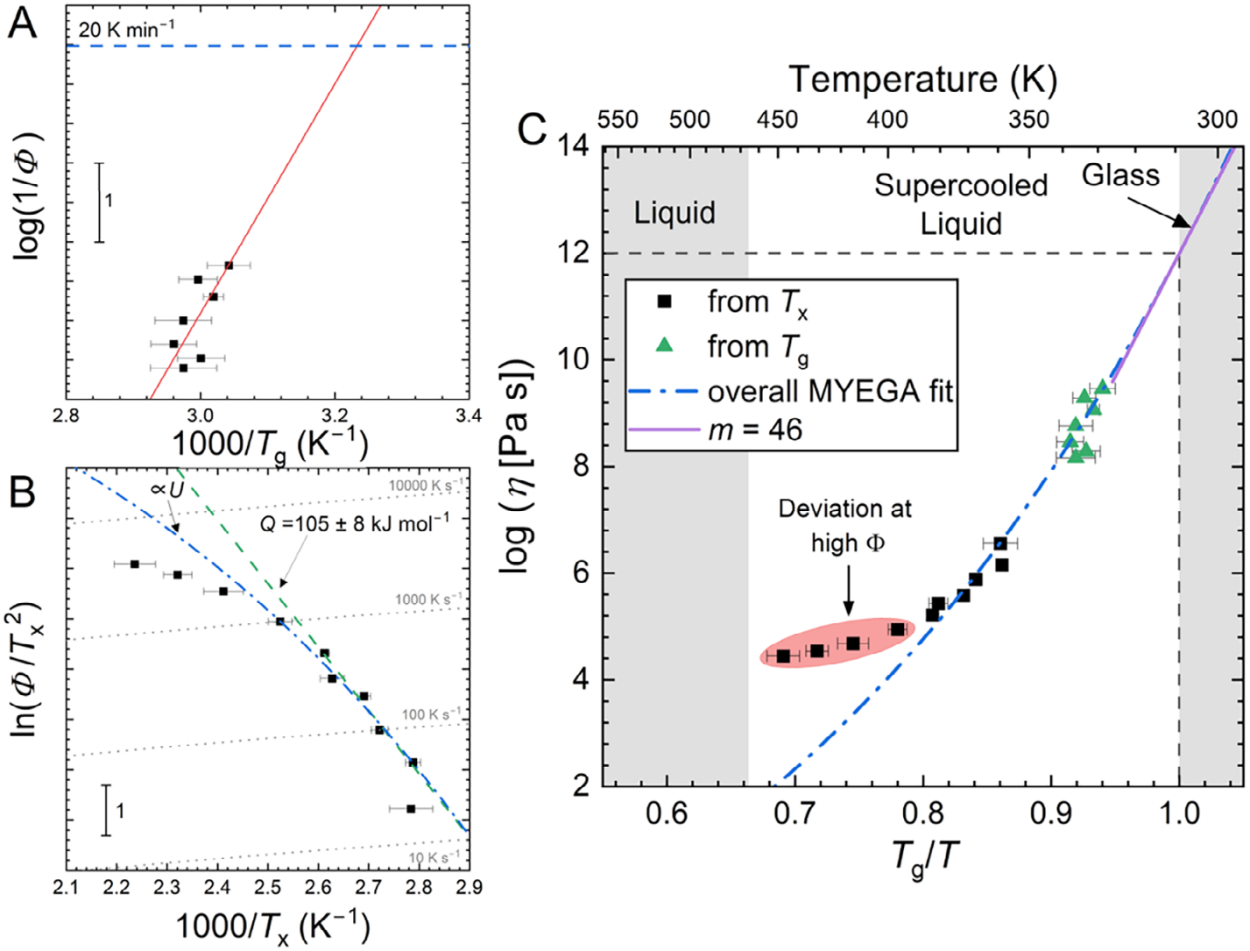
Kinetic properties of liquid (*S*‐Cl‐MBA)_2_PbI_4_. Measurements over a range of heating rate Φ used for A) Ozawa analysis of the glass transition, and B) Kissinger analysis of crystallization. An activation energy is determined from the low‐heating‐rate data. An approximate MYEGA fit to the crystal growth rate *U* is shown. C) Angell plot of the temperature dependence of viscosity estimated from the temperature dependence of *T*_x_ and *T*_g_. The data from the Kissinger plot are vertically shifted. The MYEGA fit to the combined data gives an estimate of *m* ≈ 46 for the liquid fragility.

The glass transition when heating at a conventional DSC rate of 20 K min^−1^ is taken to occur at a liquid viscosity of 10^12^ Pa s. An Ozawa plot (**Figure** **4**A) of the heating‐rate dependence of *T*_g_ measured reflects the temperature dependence of the liquid viscosity *η*(*T*), and enables extrapolation to estimate this conventional *T*_g_,^[^ ^33^ ^]^ which for (*S*‐Cl‐MBA)_2_PbI_4_ is 309 K. As no values are available from the literature, we use this estimate in our subsequent analysis. As expected, this conventional *T*_g_ is lower than the values shown at higher heating rates on FDSC thermograms; at high heating rates, the glass has less time to equilibrate with the liquid at a given temperature. The gradient of the Ozawa plot relates directly to the kinetic *fragility* that is a key descriptor of the temperature‐dependent viscosity, *η*(*T*), of the liquid.^[^ ^34^, ^35^ ^]^

We characterize *η*(*T*) further, as originally proposed for metallic glasses,^[^ ^36^ ^]^ and later applied for amorphous chalcogenides,^[^ ^23^ ^]^ by characterizing the heating‐rate dependence of *T*_x_. For (*S*‐Cl‐MBA)_2_PbI_4_, the data are displayed on a Kissinger plot of ln(Φ/*T*_x_^2^) versus (1/*T*_x_), where Φ is the heating rate (Figure 4B). Each sample was melted by heating to 550 K at 5000 K s^‒1^, then quenched at 5000 K s^‒1^, and subsequently reheated at various rates between 50 and 4000 K s^‒1^. At relatively low Φ, such plots are typically linear, the fixed slope (‒*Q*/*RT*) revealing a single‐value activation energy; in the present case, *Q* = 105 ± 8 kJ mol^‒1^. This can be compared with the similar value of *Q* = 144 kJ mol^‒1^ found for another 2D metal halide perovskite *S*‐(‒)‐1‐(1‐naphthyl)‐ethylammonium lead bromide when heated in the same range of Φ.^[^ ^19^ ^]^ As explained by Singh and Mitzi,^[^ ^16^ ^]^ however, comparison of such activation energies between different systems may not be useful. At higher Φ, the plot is curved, as seen in previous studies of glass‐forming HOIPs.^[^ ^19^ ^]^ The local gradient of the curve is dominated by the temperature dependence of the crystal growth rate,^[^ ^23^, ^37^ ^]^ and this to a good approximation is also the temperature dependence of *η* ^‒1^. The curvature of the plot thus indicates that the temperature dependence of *η* ^‒1^ is not Arrhenius. In that case, and at high Φ (exceeding roughly 10^3^ K s^‒1^), two factors affect the link between the plot and the variation of *η* ^‒1^: i) the peak of the crystallization rate no longer occurs at a fixed fraction crystallized; and ii) the crystal growth is slowed by decreased thermodynamic driving force as *T*_m_ is approached.^[^ ^23^, ^38^ ^]^ These factors partially self‐compensate, but nonetheless lead to a clear deviation in the Kissinger plot (Figure 4B). As our focus is not on the behavior at the highest Φ values, we do not attempt to eliminate the deviation by applying corrections of the kind used in earlier work.^[^ ^23^, ^38^ ^]^

The *T*_g_ data from Figure 4A and the *T*_x_ data from Figure 4B are combined in an Angell plot of log(*η*) versus log (*T*_g_/*T*) (Figure 4C).^[^ ^34^, ^35^ ^]^ The temperature dependence of each dataset should match the temperature dependence of the liquid viscosity *η* in the same temperature range. Since the Kissinger plot gives the temperature dependence rather than absolute values, the *T*_x_ data set can be arbitrarily shifted vertically to obtain the overall curve of *η*(*T*). Over a wide temperature range, *η*(*T*) is expected to follow the Mauro‐Yue‐Ellison‐Gupta‐Allan (MYEGA) form given by:^[^ ^39^ ^]^

(1) $$\eta =\eta_{0}10^{\frac{K}{T}\exp\frac{H}{T}}$$

where *K* and *H* are adjustable parameters. The pre‐factor *η*_0_ is the high‐temperature limit of viscosity, taken to be 4 × 10^−5^ Pa s. This value of *η*_0_ is also used to estimate the pre‐factor *U*_kin,0_ (Section S5, Supporting Information), for which a physically reasonable value is needed is to obtain an appropriate fit of the temperature dependence of the crystal growth rate (Figure 4B). The data in Figure 4C are fitted with *K* ≈ 780 K and *H* ≈ 570 K.

## 3 Discussion

(*S*‐Cl‐MBA)_2_PbI_4_ and (*R*‐Cl‐MBA)_2_PbBr_4_ are shown to be glass‐forming systems. The ability to form a glass in 2D HOIPs at high cooling rates is significant for many of their proposed applications. For (*S*‐Cl‐MBA)_2_PbI_4_, the critical cooling rate to avoid substantial thermal degradation or crystallization is ≈300 K s^−1^ (Figure 3A).

The reduced glass‐transition temperature *T*_rg_ (= *T*_g_/*T*_liq_), where *T*_liq_ is the liquidus temperature (the temperature at which the solid and liquid are in equilibrium), is well accepted as an indicator of glass‐forming ability (GFA), higher *T*_rg_ corresponding to higher GFA.^[^ ^40^, ^41^ ^]^ For (*S*‐Cl‐MBA)_2_PbI_4_, the conventional *T*_g_ ≈ 309 K (Figure 4A). The melting endotherm is complex, and it is not possible to accurately determine a representative *T*_liq_. We take *T*_liq_ to be in the range 450‒480 K, from which *T*_rg_ = 0.64‒0.69. This is much higher than for the phase‐change chalcogenide Ge_2_Sb_2_Te_5_, but similar to several oxide glasses and to bulk metallic glasses.^[^ ^41^ ^]^ The suggested critical cooling rate for glass formation, ≈300 K s^−1^ is similar to that of the comparable metallic glasses and higher than that of the oxide glasses.^[^ ^24^, ^42^ ^]^ Recent studies suggest the critical cooling rate for glass formation in 2D HOIPs can vary between 10^−1^ and 10^3^ K s^−1^.^[^ ^19^ ^]^

Above this critical rate, during cooling there is no detectable crystallization, but there is some thermal degradation. The sample degradation on thermal cycling (Figure 3C) shows distinct stages. At first, crystallization is hindered: the corresponding exotherm becomes smaller and shifts to higher temperature. Consequently, the melting endotherm also becomes smaller. In the second stage, the glass transition gradually decreases in intensity. TGA measurements suggest that both (*S*‐Cl‐MBA)_2_PbI_4_ and (*R*‐Cl‐MBA)_2_PbBr_4_ undergo a two‐stage degradation process (Figure S1, Supporting Information). It is reported that this multi‐stage collapse of the RP phase HOIPs is initiated by dissociation of the A‐site cations and partial release of X‐site anions from the hybrid structure. This then proceeds by decomposition of the BX_6_ octahedral layers.^[^ ^12^, ^28^ ^]^ Thus, the hindering of crystallization (Figure 3C) may be associated with the dissociation of the A‐site cations and partial iodide release; this dissociation prevents the formation of the ordered crystal structure (Figure 1). Comparing the samples used in Figure 3C and Figure S2 (Supporting Information), the first stage of degradation in the sample kept for 9 months at room temperature (Figure 3C) occurs in fewer cycles than the one measured immediately after synthesis (Figure S2, Supporting Information). The absorption of moisture during storage is known to accelerate the dissociation of A‐site cations from the hybrid structure.^[^ ^28^ ^]^ In each case, the glass transition is unaffected, in position and intensity, by the first stage of thermal degradation. The disappearance of the glass transition in the second stage of degradation may be associated with the breakdown of the BX_6_ octahedral layers due to the release of X‐site cations.^[^ ^12^ ^]^

On an Angell plot (e.g., Figure 4C) the line indicating the form of *η*(*T*) has different curvatures for different glasses. For SiO_2_, the line is nearly straight and the liquid is termed *strong*. For other systems, greater curvature of the line indicates a liquid that is more *fragile*. The form of *η*(*T*) can be characterized by the *fragility*, quantified as:^[^ ^34^ ^]^

(2) $$m={\left.\frac{d\log_{10}\eta }{d\left(\frac{T_{g}}{T}\right)}\right|}_{T=T_{g}}$$

From the MYEGA fit in Figure 4C, we estimate the (*S*‐Cl‐MBA)_2_PbI_4_ liquid to have a fragility of *m* ≈ 46. As the MYEGA form fits the data well, there is no evidence of any strong‐to‐fragile transition, as sometimes observed in other glass‐forming systems.^[^ ^43^ ^]^ The value of *m* agrees well with that (*m* ≈ 47) estimated directly from the effective activation energy of *T*_g_ in the Ozawa plot (Figure 4A).^[^ ^44^ ^]^ Comparable values for other RP‐phase 2D HOIPs (*m* ≈ 45 for (*S*)‐(–)‐1‐(1‐naphthyl)‐ethylammonium lead bromide)^[^ ^16^ ^]^ suggest that the range of *m* for HOIPS with this structure is narrow. These fragility values are similar to that of many metallic glass‐forming liquids, and lower than for organic glass‐formers (*m* ≈ 70–90) and Ge_2_Sb_2_Te_5_ (*m* ≈ 90).^[^ ^23^, ^41^, ^45^ ^]^ In contrast to these HOIPs, other types of hybrid glass‐formers such as zeolitic imidazolate frameworks, that form 3D crystalline structures, can form substantially “stronger” liquids.^[^ ^46^ ^]^

Recent work on halide perovskites^[^ ^47^ ^]^ has shown that local structure can be characterized using a combination of spectroscopic techniques. The variations in structural chemistry between 2D HOIPs of different compositions, as well as comparison with other hybrid glass‐formers remain important topics for further understanding of structure‐property relationships, and should assist the tailoring of properties for promising applications.

Knowing *T*_rg_ and *m* for 2D HOIPs enables consideration of their potential applications. Systems with high *T*_rg_ and low *m* are generally better bulk glass‐formers,^[^ ^48^ ^]^ promising for applications where functionalized HOIPs in the glassy state are attractive.^[^ ^12^ ^]^ Though processing techniques such as ball‐milling avoid the need for high cooling rates to achieve glass formation,^[^ ^21^ ^]^ increased resistance to crystallization and degradation is still preferable for their application, especially if operating temperatures are close to *T*_g_.

For phase‐change memory, fast switching is necessary and the limiting factor is the crystal growth rate.^[^ ^41^, ^49^ ^]^ Desirable fast growth is correlated with a low value of the fragility‐corrected *T*_rg_:^[^ ^41^ ^]^

(3) $$\;T_{\mathrm{gu}}=T_{\mathrm{rg}}\;-\frac{m}{505}$$

From the *T*_gu_ value, we estimate that the maximum crystal growth rate in (*S*‐Cl‐MBA)_2_PbI_4_ is three orders of magnitude lower than in Ge_2_Sb_2_Te_5_.^[^ ^41^ ^]^ Optimization would be required for this HOIP to be viable in memory applications.

For any application, it is imperative to improve the resistance to thermal degradation. Correlating the sequence of observed degradation with known mechanisms may provide guidelines for synthesis of more stable 2D HOIPs. As already noted, the resistance to crystallization appears linked to the behavior of the A‐site cation, the nature of which has been shown to affect the melting behavior of such materials.^[^ ^50^, ^51^ ^]^ Flexible X‐site linkers, with lower coordination symmetry, have also been shown to enhance mobility in the liquid;^[^ ^12^, ^52^ ^]^ in this way, they may affect the glass transition, in accord with our interpretation of the second stage of thermal degradation.

The sequence of thermal degradation is relevant for further study of glass‐forming HOIPs. Since such systems can decompose in the supercooled liquid state, the absence of a crystallization peak may be an effect of thermal degradation rather than glass formation. By giving access to higher heating and cooling rates, FDSC is well placed to isolate degradation effects. On the other hand, the necessarily small size of FDSC samples, implying a high surface‐to‐volume ratio, facilitates the uptake of moisture that accelerates the thermal degradation. To better understand the degradation and its effects on crystallization, it will be important to characterize structural and composition changes in the organic and inorganic components of 2D HOIPs. By evaluating *T*_rg_ and *m*, and identifying their relevance for functional properties and stability, the present work provides guidelines for further work.

## 4 Conclusion

Ultra‐fast (flash) differential scanning calorimetry (FDSC) shows that the 2D hybrid organic–inorganic perovskites (*S*‐Cl‐MBA)_2_PbI_4_ and (*R*‐Cl‐MBA)_2_PbBr_4_, can be quenched from the liquid into the glassy state. For (*S*‐Cl‐MBA)_2_PbI_4_, transmission electron microscopy confirms the glassy structure. The temperature dependence of the viscosity of liquid (*S*‐Cl‐MBA)_2_PbI_4_ was characterized by heating in FDSC over a wide range of heating rate (50‒4000 K s^−1^). The liquid has a fragility *m* of ≈46, similar to metallic glass‐forming liquids, but lower than for organic glass‐formers and for chalcogenides of interest for phase‐change memory. On cooling this liquid, thermal degradation occurs before the onset of crystallization. This degradation is the main barrier to reversibly forming a glass on repeated heating cycles; it occurs in two stages and can be explained in terms of the known mechanisms of multi‐stage collapse of the Ruddlesden‐Popper structure. By characterizing the reduced glass‐transition temperature *T*_rg_ and the liquid fragility *m*, the present results help to refine strategies for the synthesis of non‐volatile glass‐forming hybrid perovskites for a broad range of proposed applications. FDSC is a powerful tool for further study of these materials.

## 5 Experimental Section

### Synthesis

Following the method reported in refs. [26, 27], PbI_2_ (0.48 mmol, 221.28 mg) and *S*‐(−)‐1‐(4‐chlorophenyl)ethylamine (0.96 mmol, 132.32 µL) were dissolved in hydriodic acid (2.0 mL) at 368 K in a sealed vial. Similarly, PbBr_2_ (0.48 mmol, 221.28 mg) and *R*‐(+)‐1‐(4‐chlorophenyl)ethylamine (0.96 mmol, 132.32 µL) were dissolved in a mixture of hydrobromic acid (2.0 mL) and deionized water (4.8 mL) at 368 K. The resultant solutions were gradually cooled down to RT over a period of 2 h to precipitate a crystalline phase. The solutions were filtered to extract the precipitated crystals, which were then washed with diethyl ether and dried in vacuo.

### Thermogravimetric Analysis (TGA)

Measurements were performed using Mettler Toledo TGA2. Approximately 5 mg samples of vacuum‐dried HOIPs were placed on an alumina crucible for measurement. Data were collected under argon atmosphere from 303 to 973 K with a ramp rate of 10 K min^−1^.

### Powder X‐Ray Diffraction

Measurements were performed on a Bruker D8 ADVANCE diffractometer using CuKα radiation (*λ*  = 1.5418 Å). For each composition, measurements were performed on finely ground samples compacted into 5 mm flat plate discs. Pawley refinements were performed using TOPAS‐Academic Version 7.

### Ultra‐Fast Calorimetry (FDSC)

Measurements were made using a Mettler Toledo Flash DSC 1 equipped with UFS1 sensors. A sensor support temperature of 193 K was selected, and the chamber was purged with nitrogen gas (flow rate: 50 mL min^−1^). A small droplet of Wacker AK 60 000 Linear Silicone Fluid was placed on the chip to ensure good thermal contact with the sample. The thermogram on heating the silicone oil only was taken as a baseline, and subtracted from the thermogram of each sample measurement to determine the signal from the sample (Figure S3, Supporting Information). By measuring the Curie temperature of nickel at each heating rate, the temperature scale was calibrated to compensate for thermal lag. The sample mass was estimated from the ratio of the heat of melting measured on the initial heating ramp at 1000 K s^‒1^ to that measured in a conventional calorimeter at 20 K min^−1^ for the as‐synthesized material (∆*H*_m_ = 43.4 J g^−1^). The sample masses were kept within a narrow range (120 ± 10 ng) to lessen any effects of differing size and shape.

### Transmission Electron Microscopy

An electron‐transparent lamella was prepared from a glassy sample of (*S*‐Cl‐MBA)_2_PbI_4_ formed by quenching at 5000 K s^−1^ in FDSC. The lamella was cut using a FEI Helios Nanolab 600 focused‐ion‐beam scanning electron microscope (SEM). A thin protective layer of Pt (300 nm) was deposited over the target area using the electron beam before milling with low currents to minimize any ion‐beam exposure that may lead to amorphization or crystallization of the material. TEM investigation was performed using an FEI Tecnai Osiris, operated at an accelerating voltage of 200 kV, and equipped with a high‐brightness XFEG field‐emission gun. Diffraction patterns were recorded at a camera length of 550 mm with a Gatan UltraScan1000XP with 2048 × 2048 pixels.

## Conflict of Interest

The authors declare no conflict of interest.

## Supporting information



Supporting Information

## Data Availability

The data that support the findings of this study are available from the corresponding author upon reasonable request.
