# Supplementary material for: Glass Transition, Liquid Dynamics, and Thermal Degradation in 2D Hybrid Halide Perovskites
Source: Small. 2025 Apr 2;21(19):2500311. doi: 10.1002/smll.202500311 (PMC12067160; doi:10.1002/smll.202500311)
Supplement: Supplementary file 1 — Supporting Information [file SMLL-21-2500311-s001.docx]

Supporting Information

Glass Transition, Liquid Dynamics and Thermal Degradation in 2D Hybrid Halide Perovskites

Owain S. Houghton^#^, Chumei Ye^#^, Alison C. Twitchett-Harrison, Siân E. Dutton, Thomas D. Bennett, A. Lindsay Greer*

**S1. Crystallographic data**

**Table S1**. Crystallographic parameters from Pawley refinement of the PXRD data for (*S*-Cl-MBA)_2_PbI_4_. Reported lattice parameters are from ref. [26]. The *R*_wp_ value is a measure of quality of refinement. *R*_wp_ < 10% indicates excellent refinement of the measured data.

| *R*_wp_-value | Space Group | Lattice Parameters | Reported Lattice Parameters |
| --- | --- | --- | --- |
| 8.45% | *P*1 | *a* [100] = 8.9216(5) Å | *a* = 8.9033(5) Å |
|  |  | *b* [010] = 9.0690(3) Å | *b* = 9.0489(4) Å |
|  |  | *c* [001] = 16.3718(2) Å | *c* = 16.3438(8) Å |
|  |  | *⍺* = 86.393(3)° | *⍺* = 86.365(4)° |
|  |  | *β* = 82.192(3)° | *β =* 82.163(4)° |
|  |  | 𝛾= 89.673(3)° | 𝛾= 89.599(4)° |

**Table S2**. Crystallographic parameters from Pawley refinement of the PXRD data for (*R*-Cl-MBA)_2_PbBr_4_. Reported lattice parameters are taken from ref. [27].

| *R*_wp_-value | Space Group | Lattice Parameters | Reported Lattice Parameters |
| --- | --- | --- | --- |
| 12.68% | *P*2_1_2_1_2_1_ | *a* [100] = 7.9185(5) Å | *a* = 7.9135(3) Å |
|  |  | *b* [010] = 8.8141(12) Å | *b* = 8.8066(3) Å |
|  |  | *c* [001] = 35.5566(8) Å | *c* = 35.5777(18) Å |
|  |  | *⍺* = 90° | *⍺* = 90° |
|  |  | *β*= 90° | *β*= 90° |
|  |  | 𝛾= 90° | 𝛾= 90° |

**S2. Degradation of as-synthesized material**

**
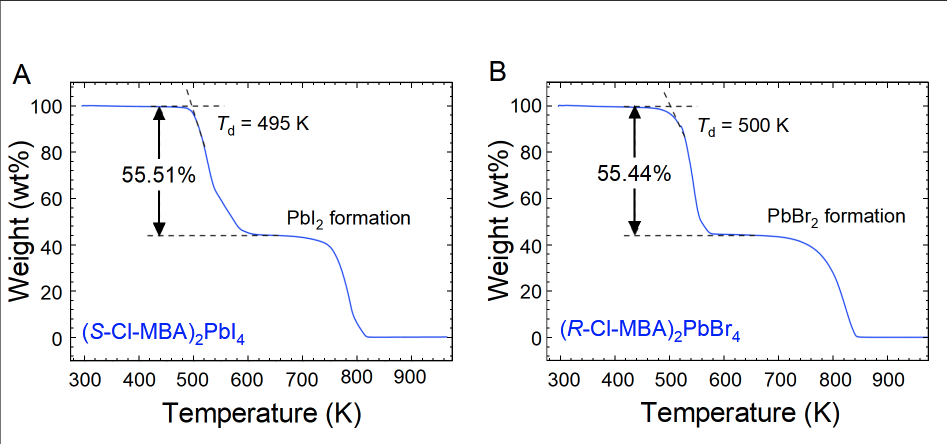
**

**Figure S1.**  TGA curves of (A) (*S*-Cl-MBA)_2_PbBr_4_ and (B) (*R*-Cl-MBA)_2_PbBr_4_. The samples were heated under argon atmosphere from room temperature at a heating rate of 10 K min^–1^. Both HOIPs show two-stage degradation.

**S3. Thermal degradation of a fresh as-synthesized sample**


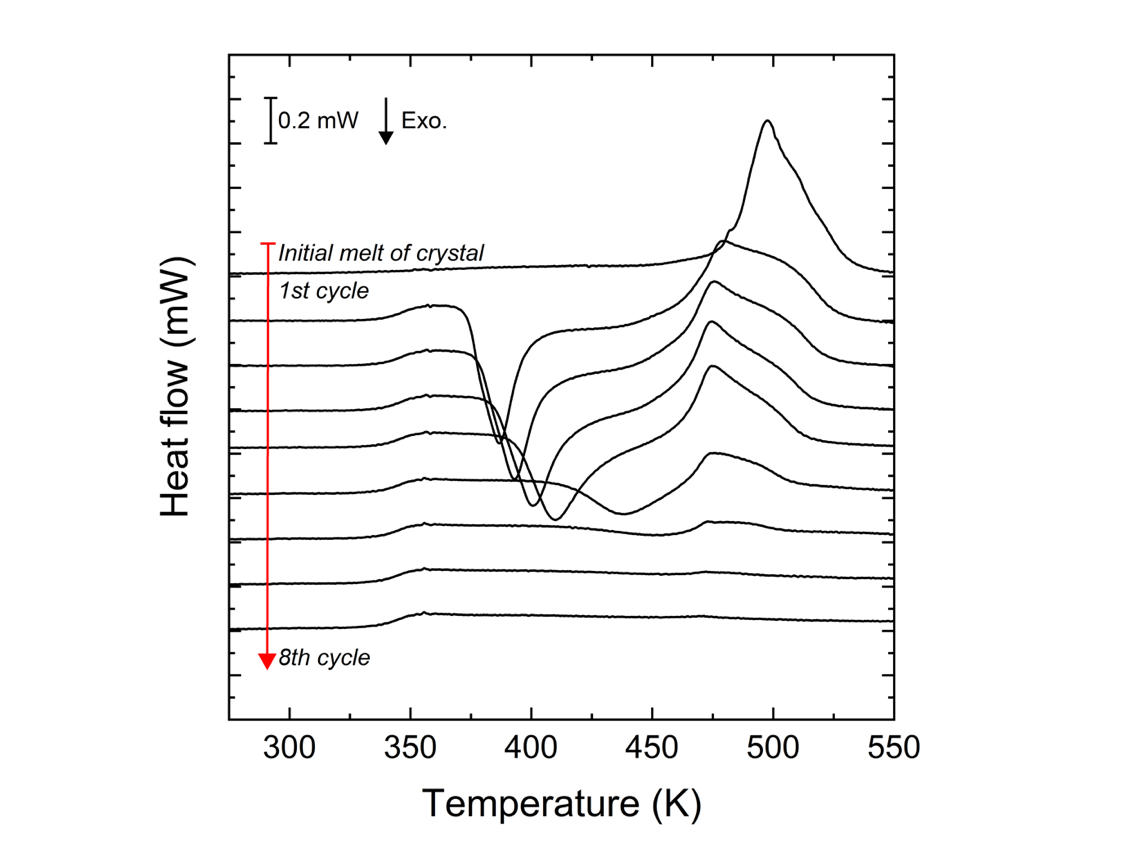


**Figure S2.**  Progressive degradation of freshly prepared (*S*-Cl-MBA)_2_PbI_4_. Thermograms of the heating segments during repeated heating and cooling at 1000 K s^‒1^ for 8 cycles. Samples were cut from material synthesized immediately before testing.

**S4. FDSC baseline subtraction**

**
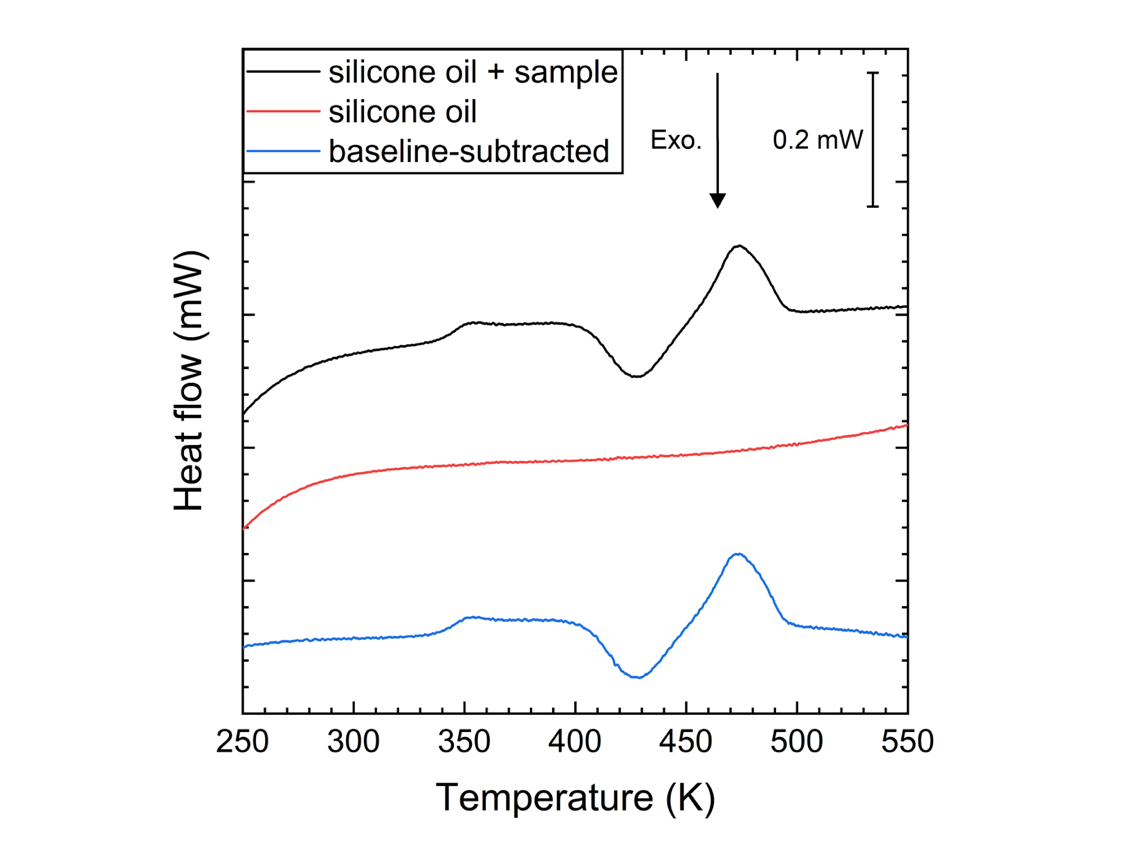
**

**Figure S3.**  For each heating protocol, a baseline curve (red) is measured with only silicone oil on the FDSC chip. The sample is then placed on top of the oil and the scan is repeated (black). The thermogram showing the signal from the sample itself (blue) is obtained by subtracting the baseline. Example shown for heating a sample of (*S*-Cl-MBA)_2_PbI_4_ at 1000 K s^‒1^ after quenching from the liquid at 1000 K s^‒1^.

**S5. Estimation of the pre-factor for crystal growth rate**

We assume that in the HOIPs there is continuous normal growth of crystals with composition matching that of the glass (i.e. polymorphic crystallization).^[53]^ The crystal growth rate *U* is then given by:

$U=U_{\mathrm{kin}}\left( 1-\exp-\frac{\Delta G}{RT} \right)$ , (S1)

where *U*_kin_ is the limiting value of *U* at high values of the free-energy driving force for crystallization ∆*G*, and *R* and *T* have their usual meanings. The temperature dependence of (*U*_kin_)^‒1^ is assumed to match that of the liquid viscosity *η*.

The variation of crystal growth rate *U* with temperature can be described by:

$U\left( T \right)=\frac{fk_{B}T}{3\pi a_{0}^{2}\eta\left( T \right)}\left[ 1-\exp\left( \frac{-\Delta G}{RT} \right) \right]$, (S2)

where *a*_0_ is the average molecular diameter and *f* is the fraction of atomic sites that are active on the liquid-crystal interface.^[54]^ Combining **Equation S1** and **Equation S2**, we arrive at an expression for the pre-factor *U*_kin,0_, in the high-temperature limit (when *η* = *η*_0_):

$U_{kin,0}^{-1}=\frac{3\pi a_{0}^{2}\eta_{0}}{fk_{B}T}$. (S3)

In the present work, we take *a*_0_ to be 1 nm, approximately that for *R*-(+)-1-(4-chlorophenyl)ethylamine, *f* to be 1 and *η*_0_ to be 4×10^‒5^ Pa s. Using this, we take *U*_kin,0_^‒1^ ≈ 0.1 s m^‒1^ as an initial refinable parameter for fitting the MYEGA model. Subsequent adjustment is in effect changing the constant of proportionality between (*U*_kin_)^‒1^ and *η*.

Additional references not cited in the main text

[53] M. D. Ediger, P. Harrowell, L. Yu, *J. Chem. Phys.* **2008**, *128*, 034709.

[54] K. F. Kelton, A. L. Greer, *Nucleation in Condensed Matter: Applications in Materials and Biology*, Elsevier, Amsterdam, Netherlands **2010**.
